# ﻿Karyotype analysis of *Quasipaaspinosa* David, 1875 (Anura, Dicroglossidae) with conventional cytogenetic techniques

**DOI:** 10.3897/compcytogen.18.116806

**Published:** 2024-06-21

**Authors:** Liaoruilin Zhang, Jianguo Xiang, Juan Li, Jie Zhou, Jinliang Hou, Yanfei Huang, Hong Li

**Affiliations:** 1 College of Fisheries, Hunan Agricultural University, No.1 Nongda Road, Furong District, Changsha, Hunan Province,410128, China Hunan Agricultural University Changsha China

**Keywords:** BrdU-banding, C-banding, karyotype, *Quasipaaspinosa*

## Abstract

The current study analyzed the chromosomal karyotype of *Quasipaaspinosa* David, 1875 from Hunan Province, China. The karyotype, C-banding, BrdU-banding pattern were characterized using direct preparation of bone-marrow cells and hemocyte cultures. The findings indicated that *Q.spinosa* was a diploid species (2n = 26) that lacked heteromorphic chromosomes and secondary constrictions. C-banding analysis revealed an abundance of positive signals in the centromere regions, while the BrdU-banding pattern showed three phases in both male and female, occurring consistently and in chronological sequence during S-phase. Notably, there was no asynchronous replication in the late phase. This study enhanced our understanding of the karyotypic structure of *Q.spinosa* by conventional cytogenetic techniques, thus providing essential scientific insights into the cytogenetics of *Q.spinosa*.

## ﻿Introduction

*Quasipaaspinosa* David, 1875 (Anura, Dicroglossidae) is an amphibian native to and widely distributed in southern and southeastern China, where it thrives in forests and hilly areas at altitudes of 500–1500 meters (Zhao 1998). *Quasipaaspinosa* holds considerable economic significance in China’s frog-breeding industry (Yu et al. 2008), as it is prized for its therapeutic and medicinal properties, together with its high reproductive rate and large populations (Lau et al. 2008). This frog species has significant nutritional and medicinal value, and has been described as the “King of a Hundred Frogs” (Mei et al. 2018). However, over the past decade, its population has declined significantly, by over 30% due to overhunting and habitat destruction. This decline has led to its being classified as endangered by the International Union for Conservation of Nature (IUCN) and the China Species Red List (Shen et al. 2015). Efforts in artificial breeding were initiated in the 1980s to meet the market demand (Chan et al. 2014).

Chromosome karyotype studies are valuable in elucidating the phylogeny of the species studied as well as assisting in its classification (Gokhman 2023) and providing an understanding of species alterations in *Q.spinosa* from a cytogenetic perspective. Regarding karyotypic research on *Q.spinosa*, Li and Wang (1983), Zheng and Hong (1984), as well as Long et al. (2021) had reported Giemsa-stained karyotypes, which consistently demonstrate a chromosomal count of 2n=26 but differing karyotypic formulas across different geographical regions.

This study presents an analysis of the karyotype of *Q.spinosa* using conventional cytogenetic techniques, aiming to contribute novel and valuable karyotypic information while enhancing the existing cytogenetic database for *Q.spinosa*.

## ﻿Material and methods

The study utilized 15 healthy adult male and 15 female *Q.spinosa*, each weighing between 90–100 g, sourced from farm located in Taiping Town (29°58'42"N, 111°05'25"E), Shimen County of Hunan Province, China. Animal treatment and the study protocol strictly adhered to ethical guidelines formulated by the Animal Protection Committee (APC) of Hunan Agricultural University (201903297) (ethics license: No. LSK 202-3-D106). Specimens were transported to the laboratory via a specialized vehicle designed to maintain breeding environment temperatures thereby reducing mortality due to temperature-induced stress.

Bone marrow-cell suspensions were prepared as previously described by Baldissera et al. (1993), with minor modifications. The animals were injected intraperitoneally with 10 μg/g of colchicine solution and allowed to rest for 4 h. They were then anesthetized with 0.05% MS-222 and bone marrow was harvested from the thighs. The harvested bone marrow was treated with 0.34% KCL solution at low osmolality for 1 h and then fixed in two changes of Carnoy’s solution, after which the cell suspension was aspirated onto the center of a slide. The slides underwent standard Giemsa staining (Howell and Black 1980), and C-banding (Sumner 1972). 20 clear images were selected from a total of 180 images captured using an Olympus BX5 digital camera. The chromosomes were classified as metacentric or submetacentric according to the criteria of Green and Sessions (1991).

The animals were anesthetized and blood was collected from the heart. The blood was injected into human peripheral blood lymphoid medium (0.2 ml of blood for every 5 ml of culture medium) which was then incubated for 90 hours in a thermostatic incubator at 28 °C in the dark. The cells were treated with 100 μg/ml bromodeoxyuridine (BrdU) 9 h before the end of the culture period, followed by 40 μg/mL fluorodeoxyuridine (FdU) for 6 h and the addition of colchicine to a final concentration of 0.03 μg/ml for 2 h. Acridine orange was added at a final concentration of 10 μg/mL 1 h before harvesting (Matsubara and Nakagome 1983). After drying, the chromosome slides were soaked in 2×SSC solution in a thermostatic water bath at 40 °C and exposed to UV light from a distance of 8 cm for 45 min. The slides were then stained with 10% Giemsa (pH = 6.8) for 15 min.

## ﻿Results

**Figure 1.**
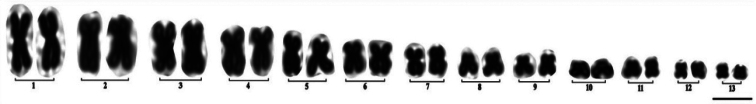
*Q.spinosa* karyotype. Scale bar: 10 μm.

**Figure 2.**
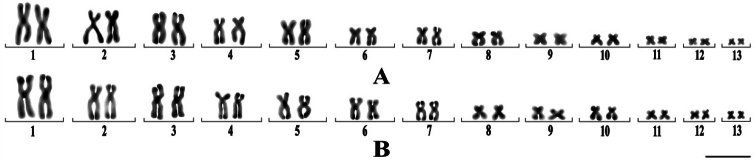
C-banding karyotypes. *Q.spinosa* male (**A**) and *Q.spinosa* female (**B**). Scale bar: 15 μm.

*Q.spinosa* exhibited a diploid number of 2n = 26 lacking heteromorphic chromosomes and secondary constrictions (Fig. 1), The chromosome pairs 2, 4, 8, and 10 were submetacentric, while the remaining chromosomes were metacentric. Table 1 shows the relative length (Guzman et al. 2022), and arm ratio, and chromosomal classification. Most chromosomes had a C-banding pattern restricted to centromere regions (Fig. 2A, B).

**Table 1.** Chromosome number (CN), relative length (RL), arm ratio (AR), and chromosomal classification (CC) of mitotic chromosome. M = metacentric chromosome; SM = submetacentric chromosome. LHV= lower and higher values for each chromosome.

CN	1	2	3	4	5	6	7	8	9	10	11	12	13
**RL**	15.38± 0.84	12.46± 0.81	11.65± 0.82	10.75± 0.62	10.04± 0.56	7.45± 0.58	6.98± 0.55	5.93± 0.55	4.97± 0.61	4.17± 0.13	3.88± 0.21	3.48± 0.11	2.96± 0.85
**LHV**	13.88~ 16.51	11.11~ 13.95	10.31~ 12.69	9.83~ 11.68	9.16~ 10.65	6.64~ 8.41	6.15~ 7.92	5.06~ 6.93	4.15~ 5.95	3.97~ 4.37	3.54~ 4.17	3.32~ 3.67	2.81~ 3.07
**AR**	1.25± 0.05	1.85± 0.12	1.50± 0.05	2.39± 0.10	1.25± 0.06	1.34± 0.05	1.34± 0.05	1.88± 0.08	1.15± 0.02	2.23± 0.07	1.28± 0.05	1.06± 0.03	1.41± 0.04
**LHV**	1.20~ 1.34	1.70~ 2.03	1.40~ 1.58	2.18~ 2.54	1.15~ 1.33	1.24~ 1.42	1.26~ 1.43	1.76~ 2.04	1.11~ 1.19	2.12~ 2.34	1.19~ 1.36	1.02~ 1.12	1.33~ 1.5
**CC**	M	SM	M	SM	M	M	M	SM	M	SM	M	M	M

After BrdU infiltration, abundant chromosomal division phases and replicative banding patterns were observed as the cell culture progressed. During the S-phase, chromosomes that had completed DNA synthesis were stained dark purplish-red or dark, while segments that were still undergoing synthesis post-BrdU infiltration appeared lavender-colored or light purplish-blue. Consequently, the replication bandings were classified into three periods based on the proportion of dark and light staining during the intermediate stage. First, in the early-replication stage at the time of BrdU treatment, the bands showed a roughly 1:1 ratio between early-replicated dark-stained bands and late-replicated light-stained bands. Second, in the mid-replication stage, dark-stained areas predominated while the chromosomes nevertheless remained distinguishable, and third, in the late replication phase, the chromosomes were almost entirely dark-stained following the completion of replication.

**Figure 3.**
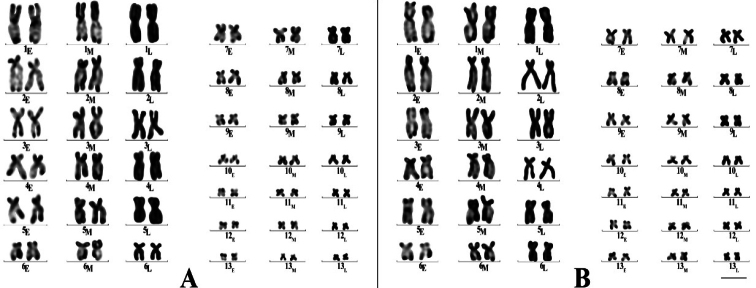
BrdU karyotypes. *Q.spinosa* male (**A)** and *Q.spinosa* female (**B**). E: early- replication phase; M: middle-replication phase; L: late-replication phase (**A, B**). Scale bar: 10 μm.

The replication timing of each of the 13 *Q.spinosa* chromosomes ranged from the very early to the late stages (Fig. 3A, B). As seen in the figure, their sequential arrangement from left to right indicated a decreasing BrdU substitution throughout the remaining S-phase. Male *Q.spinosa* displayed stronger replication bands in the early S-phase compared to the late-replication phase, while females exhibited weaker but more uniformly distributed, replication bands. Over time, these faint bands intensified, forming larger blocks. Intriguingly, the late-replication banding patterns in *Q.spinosa* suggested that neither sex exhibited heterozygosity. However, males showed more pronounced early replication banding in chromosomes 1–10 compared to females.

## ﻿Discussion

*Q.spinosa* in Hunan exhibited a karyotype comprising nine pairs of metacentric and four pairs of submetacentric chromosomes (4sm+9m), consistent with the finding reported by Long et al. (2021). However, Zheng and Hong (1984) reported a distinct karyotype for *Q.spinosa* in Fujian, characterized by seven pairs of metacentric and six pairs of submetacentric chromosomes (6sm+7m), while Li and Wang (1983) identified ten pairs of metacentric and three pairs of submetacentric chromosomes (3sm+10m) in Anhui. Furthermore, Qing et al. (2012) discovered that all 33 populations of *Q.boulengeri* Günther, 1889 were diploid with a consistent chromosome count of 2n=26 across different regions in Sichuan, China. Nevertheless, at least five distinct karyotypes were primarily observed, with notable variations occurring on chromosomes 1 and 6. Specifically, chromosome 1 was composed of either two large metacentric chromosomes (Ms) or one large M and one large telocentric chromosome (T), while chromosome 6 consisted of either two small Ms, one small M and one large subtelocentric chromosome (ST), or two large STs. These findings suggested that geographic differentiation contributed to the observed karyotype variations within the same species.

The C-banding positive region was predominantly localized in the centromere region of *Q.spinosa*, with chromosomes 1–5 exhibiting the most pronounced positivity. Moreover, C-banding positivity was also observed within the respective centromere regions of *Limnonectestaylori* Matsui, Panha, Khonsue et Kuraishi, 2010 (Phimphan and Aiumsumang 2021), *Hypsiboaspulchellus* Faivovich, Haddad, Garcia, Frost, Campbell et Wheeler, 2005 (Baraquet et al. 2013), *Ranaridibunda* Pallas, 1771 (Arslan et al. 2010). These findings collectively indicate a prevalent occurrence of C-banding across species (Kakampuy et al. 2013). The BrdU-banding findings revealed that the BrdU-banding patterns of chromosomes 1–5 in both males and females was consistent with those reported by Richard et al. (2016), with the degree change in the BrdU-banding clearly observed in all of them. Additionally, the chromosomal BrdU-banding patterns for the three stages of the S-phase resembled those described by Schempp (1981), categorized as early, middle, and late. In the present study of *Q.spinosa*, three replication stages were observed chromosomes in the S-phase for both males and females, occurring in chronological order but asynchronous replication phenomena were not observed in the late phase.

In conclusion, *Q.spinosa* was a diploid species (2n=26) with the absence of heteromorphic chromosomes and secondary constrictions. Notably, heterochromatin in the centromere region and patterns of change in the BrdU-banding were observed. In this study, the karyotypic structure of *Q.spinosa* was analyzed, providing further genetic information on *Q.spinosa*.
